# The EMBL-EBI search and sequence analysis tools APIs in 2019

**DOI:** 10.1093/nar/gkz268

**Published:** 2019-04-12

**Authors:** Fábio Madeira, Young mi Park, Joon Lee, Nicola Buso, Tamer Gur, Nandana Madhusoodanan, Prasad Basutkar, Adrian R N Tivey, Simon C Potter, Robert D Finn, Rodrigo Lopez

**Affiliations:** European Molecular Biology Laboratory, European Bioinformatics Institute (EMBL-EBI), Wellcome Trust Genome Campus, Hinxton, Cambridge CB10 1SD, UK

## Abstract

The EMBL-EBI provides free access to popular bioinformatics sequence analysis applications as well as to a full-featured text search engine with powerful cross-referencing and data retrieval capabilities. Access to these services is provided via user-friendly web interfaces and via established RESTful and SOAP Web Services APIs (https://www.ebi.ac.uk/seqdb/confluence/display/JDSAT/EMBL-EBI+Web+Services+APIs+-+Data+Retrieval). Both systems have been developed with the same core principles that allow them to integrate an ever-increasing volume of biological data, making them an integral part of many popular data resources provided at the EMBL-EBI. Here, we describe the latest improvements made to the frameworks which enhance the interconnectivity between public EMBL-EBI resources and ultimately enhance biological data discoverability, accessibility, interoperability and reusability.

## INTRODUCTION

With the advent of many technological developments, the volume and types of data generated in the life sciences have greatly expanded over the last years. High-throughput data is increasingly not only generated in fields ranging from proteomics, metabolomics and metagenomics, but also in those such as structural biology, traditionally viewed as low-throughput. This puts pressure on the data custodians that are not only expected to provide continuous access to the data, but also to do so in alternative formats and over new access methods. Application Programming Interfaces (APIs) are increasingly used to improve the way in which biological data are consumed and integrated into third-party systems. Despite these efforts, the reality is that most public data resources focus on a specific data type, but often value arises from the interconnectivity between multiple resources and datasets (1). Cross-referencing between data resources combined with expert curation and knowledge has the potential to enhance the value of the existing data. Another way of improving the value of these data is to develop novel ways to use bioinformatics applications that take advantage of these cross-references and enrich tool output and analytical results. Good examples of such applications are sequence similarity search suites such as NCBI BLAST+ (2), FASTA (3), HMMER3 (4), as well as functional classification and annotation programs such as InterProScan5 (5), which allow large collections of sequence data to be searched. The EMBL-EBI has devoted a lot of effort to develop two Web Service API-centred frameworks, Job Dispatcher (6) and EBI Search (7), for providing access to (i) sequence analysis tools and to (ii) a free text search and powerful cross-referencing engine, respectively. Here, we describe the various enhancements made recently to these services. Additionally, examples of integration between the resources are provided, which aim to demonstrate the ‘Software as a Service’ (SaaS) capabilities of the APIs.

### The frameworks

The Job Dispatcher framework (6) provides reliable Bioinformatics Sequence Analysis Web Services. Tools running under it comprise core bioinformatics analytical tools and nucleotide and protein sequence databases hosted at EMBL-EBI via three fundamental interfaces: web browser, RESTful and SOAP Web Services. The framework has three main components: (i) a tools configuration module; (ii) a cluster scheduling interface that communicates with a high-performance computer (HPC) queuing system and (iii) a rendering module for displaying enriched results. Extensive validation routines are built into the web service to ensure input parameter values and input data types sent to the tools are valid and appropriate. Similarly, tool outputs are examined to confirm tool execution, detect errors and produce human-readable reports. In many cases, visual representations of tool results are provided to help the user understand the job output both interactively using a web browser or programmatically.

EBI Search (7) is available from https://www.ebi.ac.uk/ebisearch and is a fast and scalable search engine implemented with the Apache Lucene framework (https://lucene.apache.org) that provides easy and uniform access to public biological data resources hosted at the EMBL-EBI. The search engine indexes freely available data resources in various formats such as XML, JSON and plain text from EMBL-EBI and provides text searching as well as other useful functions for query management, result filtering and cross-reference exploration through its RESTful API. This powerful API allows users to create a complex view of data resources, helping them find answers to their biological questions. EBI Search is also accessible via a web interface developed with the Angular JavaScript framework (https://angular.io/).

### Bioinformatics tools as a service

Job Dispatcher Web Services have been integrated into multiple EMBL-EBI resources. Examples of tools integration are the NCBI BLAST+ services integrated into the websites of UniProtKB (8), ENA (9) and Ensembl Genomes (10). Similar integration is done with SSEARCH (3) as part of services offered by the PDBe (11). One of the biggest users of the framework is InterPro (12) whose InterProScan 5 (5) protein sequence scanning against InterPro databases is powered by Job Dispatcher. A new integration for this update is batch HMMER3 (4) phmmer, hmmscan and hmmsearch analysis jobs, which are similarly powered by the Job Dispatcher Web Services. Dbfetch (http://www.ebi.ac.uk/Tools/dbfetch) is a service that allows a variety of data, including sequence data, to be retrieved in a variety of formats using a unique endpoint and a consistent API. It provides a web interface and programmatic interface, acting as a central support service for other EMBL-EBI services. For example, Job Dispatcher and SIFTS sequence-structure mapping provided by the PDBe (13), which also highlights the interconnectivity between data resources and the applications.

### EBI Search as a service

The capability of ‘EBI Search as a Service’ implemented in the EBI Search API has been used by more than 15 projects in EMBL-EBI (https://www.ebi.ac.uk/ebisearch/overview.ebi/about#collaborations). These include RNAcentral (14) and the OmicsDI portal (15). RNAcentral (14) generates meta-data from their comprehensive ncRNA sequence collection that is indexed by EBI Search, which creates a customised view of these data using the EBI Search API. The OmicsDI portal (15) offers integrated access to transcriptomics, genomics, proteomics and metabolomics based on 19 datasets. The search results of EMBL-EBI HMMER (4) services, as well as those of sequence similarity search results from Job Dispatcher, are enriched with links using the EBI Search cross-referencing API. The EBI Search web interface itself is a client of its own API and provides a good example of its capabilities.

**Figure 1.**
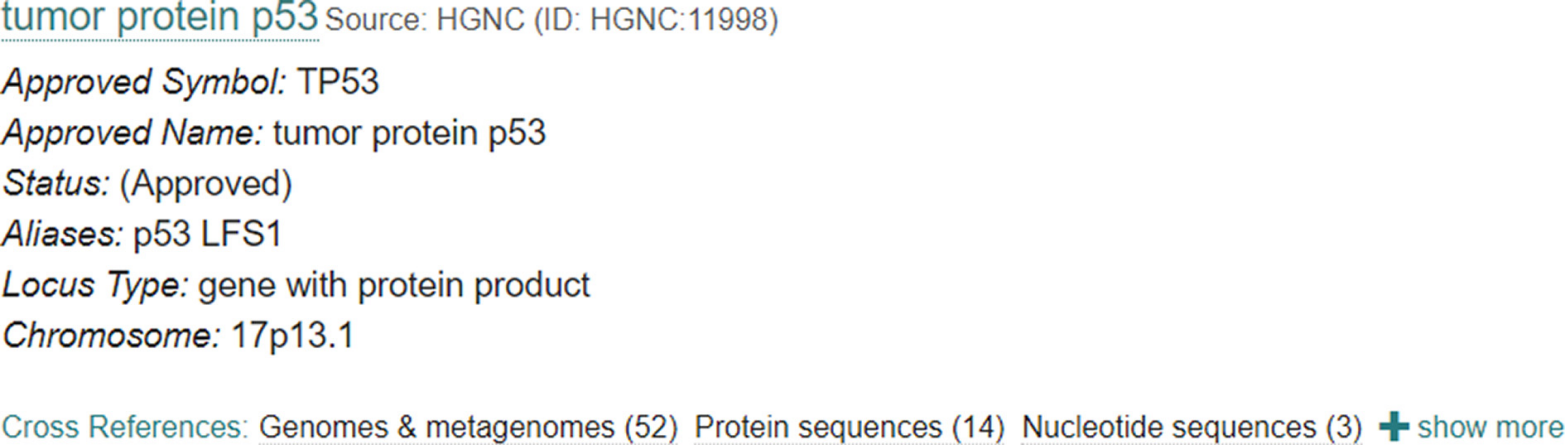
New visualization of cross-reference links in EBI Search.

The EBI Search engine indexes cross-references between entries in different domains. This is now more visible to users by displaying the number of, and links to the cross-references available. The implementation of the new visualization (see Figure 1) is the result of a collaboration with the user experience (UX) teams at the EMBL-EBI.

During 2017, EBI Search was involved in the Thor project (https://project-thor.eu/), with the aim of establishing associations between scientists and the datasets they have worked on. EBI Search is now indexing and showing these via a User Interface (UI) that scientists can use to claim datasets into their ORCID (https://orcid.org/). Further information on this service is available from: https://www.ebi.ac.uk/ebisearch/orcidclaimdocumentation.ebi.

### Updates on the frameworks and APIs

Job Dispatcher was improved with the addition of Swagger OpenAPI UI , available at https://www.ebi.ac.uk/Tools/common/tools/help. This UI covers all of the bioinformatics tools provided by Job Dispatcher and allows users to explore the API endpoints, as well as to explore valid parameters and options that each bioinformatics application expects. Collaboration with UX experts has led to improved user experience when navigating through the Job Dispatcher web pages and results. Work was carried out to improve the way tool results are displayed and how they can be integrated into workflows. Notably, the framework now provides a convenient set of example inputs, as well as outputs, for all bioinformatics tools provided. A new set of auto-generated REST clients in Python, Perl and Java have been introduced, which are actively maintained (https://github.com/ebi-wp/webservice-clients). These clients are available for local installation, but a Docker (https://www.docker.com/) container is also provided so that the clients can be reproducibly run in a variety of operating systems and environments. The Perl and Python clients include a feature that allow sequence search analysis (e.g. NCBI BLAST+, FASTA and HMMER3) to be submitted in batches, from an input that is composed by multiple sequences in fasta format (using the optional flag ‘–multifasta’). Common Workflow Language (CWL) (16) descriptions, as well as sample CWL workflows, have also been made available (https://github.com/ebi-wp/webservice-cwl) and can be used to ease the integration of the Web Services into workflows. Dbfetch now allows Cross-Origin Resource Sharing (CORS) from all origins, which enables usage of the Dbfetch API by third-party applications.

In both 2017 and 2018, various improvements to the EBI Search API have been made. A new HTTP cache mechanism has been implemented, that improves the response time on the client side and reduces the number of calls between servers and clients. As with Dbfetch, the system now allows CORS for all origins, for easy integration with third-party applications. The number of retrievable cross-references is now unlimited, thus making it possible to build a complete cross-reference network using the API. In addition to existing response formats (i.e. XML, JSON, TSV and CSV), there are new lists of ids/accession numbers that can be directly used as an input into other pipelines or workflows.

### User's privacy

The services described in this paper are General Data Protection Regulation (GDPR) compliant, which means that personal data from users, including email addresses, IPs and submitted data, are encrypted and deleted from our servers after seven days. Programmatic access to Job Dispatcher services requires the user to provide an email address, which is only used to give tailored support and guidance. Email is however optional for web browser-based usage access to the tools, which is used when provided by the user to send out a notification about the completion of the submitted jobs. In accordance with GDPR compliance, emails are not used in any other way nor distributed in any form outside of the EMBL-EBI. More information on these services GDPR compliance is provided on the EMBL-EBI website's ‘Terms of Use’ please see: https://www.ebi.ac.uk/about/terms-of-use and the service specific ‘Privacy Notice’. For the Job Dispatcher Tools described here, please see: https://www.ebi.ac.uk/data-protection/privacy-notice/sequence-analysis-and-data-retrieval-tools-job-dispatcher-and-dbfetch-, and for the EBI Search please see: https://www.ebi.ac.uk/data-protection/privacy-notice/ebi-search-support-.

### Updates on data resources and applications

In addition to updating the bioinformatics tools to the latest supported versions, new analytical tools from HMMER3 (4) phmmer and hmmscan) and from EMBOSS (17) (Dotmatcher, Dotpath, Dottup and Polydot), have been added to the Job Dispatcher in 2018, and are accessible via the APIs. Results from multiple sequence alignments (MSA) programs have been linked to launch in Jalview (18), aiming at helping users with viewing, editing and analysing their MSAs. Tools such as PromoterWise (19) and Wise2DBA (20) along with ScanProsite (21), MAFFT addseq (22) and RaXML-EPA (23) have been retired. Table 1 lists all the Bioinformatics applications currently provided with Job Dispatcher.

**Table 1.** New and updated bioinformatics tools available through Job Dispatcher in 2019. The OpenAPI user interface for these tools is available from: https://www.ebi.ac.uk/Tools/common/tools/help

Category	Tools
Multiple Sequence Alignment (https://www.ebi.ac.uk/Tools/msa/)	Clustal Omega, Kalign, MAFFT, MUSCLE, T-Coffee, MView and WebPRANK
Pairwise Sequence Alignment (https://www.ebi.ac.uk/Tools/psa/)	Needle, Stretcher, Water, Matcher, LALIGN, and GeneWise
Phylogeny Analysis (https://www.ebi.ac.uk/Tools/phylogeny/)	Simple Phylogeny
Protein Functional Analysis (https://www.ebi.ac.uk/Tools/pfa/)	InterProScan 5, PfamScan, Phobius, Pratt, RADAR, HMMER3 phmmer and HMMER3 hmmscan
RNA Analysis (https://www.ebi.ac.uk/Tools/rna/)	Infernal cmscan and MapMi
Sequence Format Conversion (https://www.ebi.ac.uk/Tools/sfc/)	Seqret and MView
Sequence Operation (https://www.ebi.ac.uk/Tools/so/)	Seqcksum
Sequence Similarity Search (https://www.ebi.ac.uk/Tools/sss/)	NCBI BLAST+, PSI-BLAST, FASTA, SSEARCH, FASTM/S/F, GGSEARCH, GLSEARCH, PSI-Search and PSI-Search2
Sequence Statistics (https://www.ebi.ac.uk/Tools/seqstats/)	SAPS, Pepinfo, Pepstats, Pepwindow, Cpgplot, Newcpgreport, Isochore, Dotmatcher, Dottup, Dotpath and Polydot
Sequence Translation (https://www.ebi.ac.uk/Tools/st/)	Transeq, Sixpack, Backtranseq and Backtranambig

New sequence databases have been added to Job Dispatcher, which are available for Sequence Similarity Searches. These include MEROPS (24) databases, ChEMBL (25) targets, UniProtKB Reference Proteomes (8), as well as protein family databases used by HMMER3 (4). IPD-HMC, IPD-KIR and IPD-IHMGT/HLA (26) databases were also updated so that nucleotide sequences are split into CDS (coding sequence) and Genomic sequences. Table 2 lists all the databases currently provided in Job Dispatcher.

**Table 2.** New and Updated Data resources available through Job Dispatcher in 2019

Category	Datasets
UniProtKB protein sequences	UniProtKB, SwissProt, SwissProt Isoforms, TrEMBL, UniProtKB Taxonomic Subsets (13 subgroups, including: bacteria, archaea, eukaryota, etc.), Reference Proteomes, Representative Proteomes (15, 35, 55, 75), UniProt Reference (UniRef 50, 90 and 100), UniParc, Unimes and UniProtKB-PDB
Patent protein sequences	EPO, JPO, KIPO, UPSPTO
Structures protein sequences	PDBe and PSI structure targets
Protein families	Pfam, TIGRFAM, Superfamily, Gene3D, PIRSF and TreeFam
Other protein sequences	Enzyme Portal, IntAct, IPD-IMGT/HLA, IPD-KIR, IPD-MHC, MEROPS (MP, MPEP and MPRO), ChEMBL and Quest for Orthologs
ENA nucleotide sequences	ENA sequence releases and updates for Coding, Non-coding, Barcode, Geospatial and others (10 subgroups, including: Expressed Sequence Tag, Genome Survey Sequence, etc.)
Ensembl Genomes sequences	Genomes from Bacteria, Fungi, Plants, Metazoa and Protists
Structures of nucleotide sequences	PDBe
Other nucleotide sequences	IMGT/LIGM-DB, IMGT/HLA (CDS and genomic), IPD-KIR (CDS and genomic) and IPD-MHC (CDS and genomic)

The data resources available in EBI Search are grouped by biological categories (Table 3). Since the last update, Europe PMC (27) (enriched replacement of Medline), BioSamples (28), Rfam (29), reviewed ChEMBL (25), OLS (30), dbGaP (31), EVA (32), InterPro 7 (12) and bio.tools (33) (replacement of ELIXIR registry) have been added as new resources. PomBase (34), MEDLINE and the ELIXIR registry have been retired.

**Table 3.** Data resources available through EBI Search in 2019

Category	Data resources
Genomes and metagenomes	Ensembl Genomes, Ensembl, HGNC, DGVa, EGA, LRG, WormBase ParaSite, MGnify
Nucleotide sequences	ENA, RNAcentral, Rfam, NRNL1, NRNL2, IMGT/HLA, IPD-KIR, IPD-MHC
Protein sequences	UniProtKB, UniParc, UniRef, EPO, JPO, KIPO, USPTO, NRPL1, NRPL2
Macromolecular structures	PDBe, EMDB
Bioactive molecules	ChEBI, ChEMBL, Ligands
Gene expression	ArrayExpress, Expression Atlases, GEO, dbGaP
Molecular interactions	IntAct
Reactions, pathways	Rhea, Reactome, BioModels, MetaboLights, MetabolomeExpress, Metabolomics Workbench
Protein families	InterPro, TreeFam, Pfam, MEROPS, GPCRDB
Protein expression data	PRIDE, GNPS, GPMdb, MassIVE, PeptideAtlas, LINCS, Paxdb, jPOST
Enzymes	IntEnz, Enzyme Portal
Literature	Europe PMC, Patent families
Samples and ontologies	Taxonomy, GO, EFO, SBO, MESH, BioSamples, Identifiers.org registry, ORCID data claims, OLS, bio.tools
Diseases	OMIM, Human diseases

### Examples of using the apis

A number of clients and docker images are available from https://github.com/ebi-wp/webservice-clients for easily accessing the services. Below are some basic examples using the Job Dispatcher APIs for the Clustal Omega tool:

**Submit a job to the Clustal Omega service using dbfetch to obtain sequences**:

curl -X POST –header ‘Content-Type: application/x-www-form-urlencoded’ -d ‘stype=protein&sequence=uniprot:wap_rat,uniprot:wap_mouse,uniprot:wap_rabit, &email=<youremail here>’ ‘https://www.ebi.ac.uk/Tools/services/rest/clustalo/run’

The above returns a string, which corresponds to the job identifier or **jobID**,which has the following form: clustalo-I20190408-092628-0974-9944177-p1m

**Check the status of a job:**

curl ‘https://www.ebi.ac.uk/Tools/services/rest/clustalo/status/<jobID>’

**Check which result types are available:**

curl –header ‘Accept: application/xml’ ‘https://www.ebi.ac.uk/Tools/services/rest/clustalo/resulttypes/<jobID>’

**Get the alignment output:**

curl –header ‘Accept: text/x-clustalw-alignment’ ‘https://www.ebi.ac.uk/Tools/services/rest/clustalo/result/<jobID>/aln-clustal_num’

Similarly, below are some simple examples of using the EBI Search API:

**Do a search across all domains for ‘BRCA1’:**

curl –header ‘Accept: application/xml’ ‘http://www.ebi.ac.uk/ebisearch/ws/rest/?query=brca1’

**Find all domains having associations with a given UniProt entry (BRCA1_HUMAN):**

curl –header ‘Accept: application/xml’ ‘http://www.ebi.ac.uk/ebisearch/ws/rest/uniprot/entry/brca1_human/xref’

**Given the UniProt entry for BRCA1 product, find and display the associated entry or entries in Ensembl:**

curl –header ‘Accept: application/xml’ ‘http://www.ebi.ac.uk/ebisearch/ws/rest/uniprot/entry/brca1_human/xref/ensembl_gene’

**Also, it is possible to find the UniProt entry from the associated entries of ensembl_gene by the bi-directional cross-reference:**

curl –header ‘Accept: application/xml’ ‘http://www.ebi.ac.uk/ebisearch/ws/rest/ensembl_gene/entry/ENSG00000012048/xref/uniprot’

Further details and examples about how to use these APIs can be found at: https://www.ebi.ac.uk/Tools/common/tools/help and https://www.ebi.ac.uk/ebisearch/swagger.ebi

### Usage statistics

Over 2017 and 2018, Job Dispatcher services have seen a continuous increase in usage. In 2017, ∼140 million jobs were performed, from over 900,000 unique IPs worldwide. In 2018, the number of jobs increased to 146 million. Usage through the website accounted for 7.6%, whereas REST programmatic access accounted for 88.2% and 4.2% via the SOAP interface.

Similarly, there has been continuous growth in traffic to EBI Search. The number of requests was about 282 million in 2017 and 550 million in 2018. In 2017 and 2018, ∼295 000 and 311 000 unique IPs, respectively, accessed the search system from across the globe.

## DISCUSSION

Job Dispatcher and EBI Search are core services used extensively by other resources at the EMBL-EBI, and collaboration between these two projects is essential. The use of cross-references provided in the results of sequence similarity search tools is a good example of this collaboration. Further work is underway to add functions from EBI Search in the Job Dispatcher sequence similarity search tools, such as faceting to give users the ability to filter matches by, e.g. taxonomy status, keywords, GO Terms, etc. Importantly, efforts involving all the teams using these services, on improving the synchronicity between data releases, are being made. Future work plans for the Job Dispatcher framework include extending the usage of CWL to improve integration of the services into analysis pipelines and workflows. This will allow for an enhanced interoperability between the various tools as well as the data. In addition, development will focus on overhauling the entire frontend to use modern JavaScript frameworks. Interactive graphics will also be included to improve the display of common tool outputs, such as multiple sequence alignments, phylogenetic trees and protein three-dimensional structures.

On the EBI Search front the focus is on continued addition of features in response to user feedback and further relaxation of existing constraints to give more search power to the users. Further investment is being made to maintain the scalability and stability of the search system. Upgrading major software libraries and removing software and data legacy is high on the work agenda, as is migrating to the latest storage and compute infrastructures, improving service development and delivery using modern technologies (e.g. GitLab (https://gitlab.com), Docker (https://docker.com), Kubernetes (https://kubernetes.io/)).

## DATA AVAILABILITY

The Bioinformatics Tools are accessible from https://www.ebi.ac.uk/services. EBI Search is available from https://www.ebi.ac.uk/ebisearch or from many pages on the EMBL-EBI's web site. Sample Web Service Clients as well as CWL workflows are available in the following GitHub repositories: https://github.com/ebi-wp/webservice-clients, and https://github.com/ebi-wp/webservice-cwl, respectively.
